# The impact of Plan S on scholarly journals from less developed European countries

**DOI:** 10.3325/cmj.2021.62.4

**Published:** 2021-02

**Authors:** Jelka Petrak, Lea Škorić, Bojan Macan

**Affiliations:** 1 Central Medical Library, University of Zagreb School of Medicine, Zagreb, Croatia *lea.skoric@mef.hr*; 2 Centre for Scientific Information, Ruđer Bošković Institute, Zagreb, Croatia

In September 2018, Science Europe (*https://www.scienceeurope.org/)* launched the cOalition S initiative for increasing open access (OA) to research data and publications derived from publicly funded research projects. The backbone of the initiative is Plan S, with one main goal: “*With effect from 2021, all scholarly publications on the results from research funded by public or private grants provided by national, regional and international research councils and funding bodies, must be published in Open Access Journals, on Open Access Platforms, or made immediately available through Open Access Repositories without embargo*” (1). Whichever of these three routes is taken, “*all publications must be published under an open license, preferably the Creative Commons Attribution license (CC BY), in order to fulfil the requirements defined by the Berlin Declaration*.” Plan S defines OA platforms as publishing outlets for original research publications (such as Wellcome Open Research or Open Research Europe, which will soon be launched by the European Commission), and not those that are serving to aggregate or re-publish content already being published elsewhere. It recognizes the importance of the green route to OA and strongly encourages the deposition of all publications in a repository, irrespective of the chosen route. Plan S recommends not support hybrid journals in their current form. Instead, it encourages various transformative agreements with publishers of subscription journals for their transition to fully OA journals by gradual increment of their OA content and by offsetting subscription income from payments for publishing services to avoid double payments (2). For example, the ESAC Transformative Agreement Registry has compiled a list of more than 160 transformative agreements signed all over the world between large scientific publishers and consortia/institutions (3). Plan S underlines that all OA publication fees would be covered by the funders. Though primarily focused on scholarly articles, cOalition S plans to provide recommendations for monographs, book chapters, and research data in the near future as well.

Plan S has been supported by more than 20 European funding bodies and research councils, as well as by some charitable and international funders and research organizations (4). While new members are still joining, some are withdrawing their support, such as the European Research Council (ERC), which in July 2020 decided to follow an independent path toward OA. The ERC Scientific Council considers it detrimental that the plan of cOalition S does not to support publishing in hybrid journals outside the transformative agreements, especially for early career researchers, researchers working in countries with fewer funding opportunities, or working in the fields in which open access policies are more difficult to implement (5).

## Challenges for the implementation of Plan S

The coronavirus disease 2019 (COVID-19) crisis has shown that OA is the one and only solution allowing immediate access to relevant information on new research developments. The pandemic had “*motivated even the big commercial scientific publishers*” to provide free and immediate access to all publications and data related to COVID-19 (6). Will this experience really motivate commercial publishers to accept the OA principles in the way that Plan S recommends? Some stakeholders assume that the most influential journals will continue to function as subscription or hybrid journals, and that the scientists working in countries following Plan S, notably in Europe, will be handicapped in the competition with scientists working in countries outside cOAlition S, for example in the US or Asia (7).

A European publisher reasoned that a plan driven by direct payments from grants is incompatible with the disciplines and sub-fields where there is no direct grant funding, eg, humanities and social sciences (HSS). Many of these disciplines have a more national focus and receive scarcer grant funding (8). An analysis of the Directory of Open Access Journals (DOAJ) revealed that a majority of included journals did not charge article processing, and that most of them were HSS journals from small publishers. The same is true for publishers with one or two journals in the fields of science, technology, engineering, and medicine (STEM). Most of them do not charge article processing. However, larger STEM publishers are financing their activities mostly through article processing charges (APC) (9).

At the same time, an analysis by the Institute of Scientific Information showed that in 2017 the research supported by Plan S initial funding bodies yielded only 6.4% of papers indexed in the Web of Science databases (10). These papers, however, are well cited and published in high-impact journals often held by major publishing houses with high APCs.

Since researchers from different countries have radically different access to funds to pay APCs, some rely heavily on subscription journals, where they do not have to pay any charges to publish their work.

The main challenges set before publishers under Plan S are as follows:

• Establishing fair and reasonable prices for publishing services, including waiver policies as well as implementation of caps if unreasonable price levels are observed (11);

• Improving APC transparency (see, for example, *https://blog.f1000.com/2020/08/19/price-transparency-on-f1000research/)*

• Lack of financial support for hybrid journals. This might provoke a strong reaction by “big” publishers;

• Increased technical requirements for all publication venues;

• Obligatory CC licenses.

## The impact of Plan S on less developed (European) countries

A recent study of national or regional influences on OA publication culture (12) suggests that economically less developed countries with relatively low research and development budgets directly or indirectly support open access to their research outputs. Joining cOAlition S and compliance with Plan S OA publishing model, however, could pose an insurmountable challenge, considering that national research funders in these countries are already struggling with budgets they have. Academic journals in these countries are not-for-profit by default, as they are being published by learned societies, universities, or research institutes. These publishers usually release just one or a couple of journal titles covering the fields and promoting the mission of their parent institutions. Publishing in OA is their way to ensure wider visibility and/or impact in the global research community.

However, the publishing model proposed by Plan S mainly aims at paywall journals and different models of transformative agreements. It does not include non-APC funding models, and their survival will probably continue to depend on the local situation. DOAJ includes, for example, 351 Romanian, 126 Croatian, and 70 Bulgarian journals. Their “diamond” OA publishing model (free access for readers and article processing for authors) is supported entirely by public funds and revenues (13-15). One may wonder how many of these titles actually publish original research and whether the research output of these countries really requires that many titles. The competition for public funding in these countries has intensified over the last two decades, as allocated revenue cannot keep up with the demand to cover even the basic expenses of all journals, which leaves them at the mercy of current government policies and strategies of scientific development and alternative avenues of income, which are scarce.

The prospective of losing financial support from governments/institutions may soon drive diamond OA journals, especially those in the STEM area, to embrace the APC model to survive. Many local STEM journals whose mission is not primarily to publish new research even face extinction. Humanities and partly social sciences journals are in a somewhat better position, as they are more likely to receive national support and keep the diamond OA model because of their national/local outreach.

The subscription-based non-OA publishing model is not an option for these small journals/publishers, as their subscriber base is too small to ensure self-sustainability. This is one of the reasons why a number of small not-for-profit publishers have already accepted offers from large publishers to become part of their journal portfolio (16).

To sum up, even the basic Plan S requirements for OA journals, publishing platforms, and OA repositories may be out of reach for many small publishers and/or institutions that maintain their digital repositories. Publishers from countries with centrally financed and developed national OA platforms, such as Croatia (17), are in a slightly better position.

## What about medicine?

The key point of publishing medical research – to immediately share results without delay or restrictions for the sake of public interest – coincides with the main principles of OA. Public health and clinical medicine are among the fields with the fastest growth of OA publications (18). A common global OA infrastructure for biomedicine was established more than 20 years ago by the US National Library of Medicine. Medline has been freely available at the PubMed platform since 1997, and PubMed Central (PMC), a free full-text repository of biomedical journal literature, has been serving the biomedical community since the 2000s. Publishers' participation in PMC is voluntary, but journals have to fulfill certain scientific and technical standards (https://www.ncbi.nlm.nih.gov/pmc/about/guidelines/#techqual). As the number of publications grew during the COVID-19 pandemic at an unprecedented rate, and the immediate free access to relevant scientific information became of utmost importance, preprint servers and free access to research data also became increasingly important (19). But, there are many concerns regarding unsupervised re-use of data or publication of results without editorial review in the absence of quality control on study design and statistics (20). Keeping editorial processes and decisions in medical journals completely independent of authors’ and funders’ interests is now more important than ever (21).

According to the results of a recent study on Croatian OA publications (22), 53.5% of all OA papers by Croatian authors have been published in local, Croatian journals. However, the bulk of these are humanities and social sciences papers. At the same time, only 8.5% of all natural sciences papers and 15.6% of all medical and health sciences papers by Croatian authors were published in Croatian journals. These numbers show that STEM authors are mainly oriented to international journals and international cooperation.

Many local medical journals aim predominantly at local health problems and local pathology. Their role in physicians’ and other health professionals’ continuing education is also important, as well as their efforts in developing and maintaining the standards of local biomedical nomenclature. Their number will probably decrease, but in the near future they will maintain their position. Their funding could come from local resources, not necessarily from the government.

Medical journals with international aspirations could find themselves in a more difficult situation. If Plan S should favor the intrinsic quality of an OA publication over its bibliometric accomplishments or publisher reputation, the place of publication would, in theory, be less important. In practice, however, it seems improbable that peripheral journals would be receiving an increasing flow of quality submissions. Even if these journals meet technical requirements (metadata, DOIs, long-term preservation, etc), the “brand-name” core journals will always be preferred. So, the question is whether these peripheral STEM journals will become an outlet for papers rejected by the core journals and whether they will attract authors with scarce grant money.

## Conclusion

Many European research funders have been endorsing cOAlition S from the very beginning and supporting Plan S as one of the paths to science without publication paywalls. Its aims, principles, and scope are a step forward to open science. Despite this boost, it remains to be seen whether Plan S will have unexpected long-term effects on journals from small or peripheral scientific communities. Many researchers in these communities mainly publish in their domestic journals. Despite limited international reception, these journals can play an important role by increasing access to locally relevant information and helping domestic researchers to improve their publication performance. If the diamond OA publishing model of these journals loses stable national financial support, it would be an additional blow to small scientific communities.
